# Motor Timing and Covariation with Time Perception: Investigating the Role of Handedness

**DOI:** 10.3389/fnbeh.2017.00147

**Published:** 2017-08-15

**Authors:** Louise O’Regan, Michiel M. Spapé, Deborah J. Serrien

**Affiliations:** ^1^School of Psychology, University of Nottingham Nottingham, United Kingdom; ^2^Department of Psychology, Liverpool Hope University Liverpool, United Kingdom

**Keywords:** handedness, individual differences, sensorimotor timing, time production, time estimation

## Abstract

Time is a fundamental dimension of our behavior and enables us to guide our actions and to experience time such as predicting collisions or listening to music. In this study, we investigate the regulation and covariation of motor timing and time perception functions in left- and right-handers who are characterized by distinct brain processing mechanisms for cognitive-motor control. To this purpose, we use a combination of tasks that assess the timed responses during movements and the perception of time intervals. The results showed a positive association across left- and right-handers between movement-driven timing and perceived interval duration when adopting a preferred tempo, suggesting cross-domain coupling between both abilities when an intrinsic timescale is present. Handedness guided motor timing during externally-driven conditions that required cognitive intervention, which specifies the relevance of action expertise for the performance of timed-based motor activities. Overall, our results reveal that individual variation across domain-general and domain-specific levels of organization plays a steering role in how one predicts, perceives and experiences time, which accordingly impacts on cognition and behavior.

## Introduction

Time is a fundamental dimension of our behavior and plays an essential role in guiding everyday activities such as predicting collisions when determining whether it is safe to cross the road, clapping to the beat of a song or applauding a performance at a concert (Néda et al., 2000; Spapé and Serrien, 2011). This demonstrates the automatic nature of timed responses to a stimulus or event; a basic skill that has formed the basis of sensorimotor synchronization research (Aschersleben, 2002; Repp and Su, 2013). In particular, a common experimental setup requires participants to produce a sequence of finger taps to an external pacing signal such as tones. Usually, taps occur about 20–80 ms before the onset of the tones, demonstrating an anticipatory asynchrony when the external pacing signal is regular (Aschersleben, 2002; Stenneken et al., 2002; Serrien and Spapé, 2010). This suggests that participants predict the timing of upcoming events. To achieve optimal synchronization, two types of error correction exist that reduce the asynchrony that arises due to noise or timing irregularities. Whereas phase correction is an automatic adjustment that depends on the intention to maintain synchronization based on the previous tap-tone asynchrony, period correction represents an intentional modification that also involves attention and awareness of tempo change (Repp, 2005). In addition to processes of anticipation that index the prediction of events, processes of adaptation occur that assist the reaction to events. Both types of processes recruit a mixture of automatic sensorimotor and conscious cognitive operations (Pollok et al., 2004; Pecenka et al., 2013; van der Steen and Keller, 2013).

Not all timed behavior relies on external signals. In particular, during internally-driven activities, individuals spontaneously adopt a preferred tempo (Collyer et al., 1994; Delevoye-Turrell et al., 2014). In experimental settings, this type of activity can be investigated by means of self-paced tapping tasks that enable participants to freely select their most comfortable rate, relying on internal pacing due to the absence of external cues. A natural tempo is confined by lower and upper boundaries as it is difficult to infinitely perform fast repetitive movements that become uncontrollable or slow repetitive movements that become perceived as a series of discrete components. Fraisse (1982) underlined that a tempo of about 600 ms is most representative for spontaneous motor activities although individual differences exist. However, a preferred tempo is a hard-wired characteristic of the motor system as it operates across repeated motor actions such as walking, clapping or finger tapping (van Noorden and Moelants, 1999).

The dimension of time is not only essential for motor skills but also in the context of time perception by which people structure their mental time experiences such as keeping appointments (Harrington et al., 1998; Meck and Benson, 2002; Merchant et al., 2013). Time perception comprises at least two main sources: judgement of the passage of time and evaluation of interval duration. Whereas the passage of time is usually assessed by questionnaire, the evaluation of time intervals can be quantified through tasks that involve time production (to produce the duration of intervals) and time estimation (to estimate the duration of specified intervals) performed in paradigms during which participants establish present-time (prospective) or past-time (retrospective) judgements (Block, 1990). Noteworthy is that the subjective experience of interval duration is modulated by factors that are independent of physical time. In particular, a time interval can be perceived differently across individuals due to psychological states or personality traits (Weiner et al., 2016). In addition, contextual factors such as arousal and attention induce subjective time distortions (Coull and Nobre, 2008). For example, inattention to time causes an interval to be perceived as shorter whereas arousal triggers an event to be perceived as longer.

Previous neuroimaging and neuropsychological research have revealed that a distributed network of frontal regions including premotor and prefrontal correlates in addition to non-frontal regions such as the basal ganglia, cerebellum and parietal areas facilitate the performance of timing skills (Harrington et al., 1998; Spencer et al., 2003; Buhusi and Meck, 2005; Lewis and Miall, 2006; Coull et al., 2011). However, the brain areas that process timing-related information are flexibly integrated as a result of the existing constraints (Witt et al., 2008). Therefore, internal and external factors can trigger an overlap of processing resources that lead to the coupling of timing skills. In this respect, motor timing and time perception can mutually influence one another such that repetitive actions help to calibrate interval timing as established from a dual-tasking paradigm (Carlini and French, 2014).

In this study, we investigate the relationship between the timing abilities that guide our actions and those that allow us to experience time. A covariation could be viewed as evidence for common mechanisms that support the ability to time responses across domain. However, timing skills are subject to individual differences to the extent that people markedly differ in their anticipatory abilities of events (Pecenka and Keller, 2009) as well as in the preferred tempo they adopt (Fraisse, 1982). This is in agreement with research that has demonstrated individual variation in behavior and brain representations for a range of traits such as decision-making (Mériau et al., 2006; Buda et al., 2011; Gilaie-Dotan et al., 2011; Hu et al., 2014; Eres et al., 2015). In this respect, one important trait that drives individual differences is handedness, which reflects a bias towards the preferred hand for skilled unimanual and bimanual activities (e.g., writing, throwing a ball or peeling an apple). Asymmetrical hand use is not only observed at the behavioral level but is also reflected at the neural level. In particular, changes in sensorimotor and cognitive control are noticed as a function of handedness with left- and right-handers showing distinct processing mechanisms within as well as between hemispheres (Willems and Hagoort, 2009; Beratis et al., 2010; Serrien et al., 2012; Pool et al., 2014; Reid and Serrien, 2014). We know little about how internal and external dimensions influence timing processes. Here, it is hypothesized that individual factors and contextual situations guide motor timing and time perception.

## Materials and Methods

### Participants

A total of 38 healthy individuals who were all involved in University level education participated in this study (Table 1). They reported no neurological or psychiatric illnesses as evaluated by a standardized questionnaire, and had normal or corrected-to-normal vision. Participants provided written informed consent prior to the start of the experiment in accordance with the declaration of Helsinki, and were reimbursed for their participation. The study was approved by the ethics committee of the School of Psychology, University of Nottingham.

**Table 1:** Main demographic details of the left-handed (LH) and right-handed (RH) participants.

Participants	LH	RH
Number (N)	19	19
Male/female (N)	7/12	6/13
Music training (N)	2	3
Age (mean ± SD, years)	21.0 ± 6.2	22.8 ± 8.5
LI (mean ± SD)	14.9 ± 10.8	94.1 ± 6.4

*LI, laterality index*.

### Questionnaires

A handedness questionnaire was used to obtain details about the participants’ handedness (Supplementary Material, Appendix 1). The questionnaire consisted of 20 questions about hand preference for unimanual activities (such as for writing) and bimanual activities (such as for opening the lid from a drink can, Nicholls et al., 2013). Noteworthy is that self-classification as a left- or right-hander was the same as classification according to writing hand. The questionnaire made use of a 5-point Likert scale that ranged between always left, usually left, equal, usually right and always right. Using this response format, the score per item was calculated by giving a value of 0 to always left, 1 to usually left, 2 to both equally, 3 to usually right and a value of 4 to always right. Subsequently, the scores of all items were added for each participant, divided by the maximum score of the questionnaire and multiplied by 100. This provided a laterality index (LI) for each participant that ranged from 0 (extreme left-handedness) to 100 (extreme right-handedness) and resulted in 19 left-handers (LI: 14.9 ± 10.8) and 19 right-handers (LI: 94.1 ± 6.4). Eight of the left-handers were consistent handers (LI: 5.0 ± 2.4) whereas eleven were classified as inconsistent handers (LI: 22.1 ± 8.4). One of the right-handers was labeled as an inconsistent hander (LI: 73). Consistent left-handers/right-handers were those who performed the activities always/usually with their left/right hand, while inconsistent handers were those who used their non-preferred hand always for at least one activity and usually for at least two or more activities. In addition to the handedness questionnaire, three questions about foot preference were asked and using a 5-point Likert scale revealed a footedness score (total score for each participant, divided by the maximum score and multiplied by 100) of 25.9 ± 11.3 which reflected left-footedness for left-handers and 74.0 ± 15.1 which referred to right-footedness for right-handers.

The time questionnaire involved 13 questions about the passage of time and assessed whether individuals experienced time as passing slowly or quickly (Supplementary Material, Appendix 2). Moreover, the questions reflected subjective feelings of life experiences (Lamotte et al., 2014). First, questions involved effects of recent life changes since it is thought that more activity gives the impression that time is moving at a fast pace. Second, questions associated with the amount of time pressure, routine management and rushing experiences in life as feelings of being busy or not having enough time to complete activities reflects the sensation that time is fleeting. We used a 3-point Likert scale by giving a value of 1 to disagree, 2 to neutral, and 3 to agree such that higher/lower ratings indicated an accelerated/decelerated subjective time passage. Accordingly, we calculated a time questionnaire index, taking into account the type and total score of all items divided by the maximum score and multiplied by 100. This score was analyzed by means of an independent *t*-test as a function of handedness, which showed no significant effect, *t*_(36)_ = 0.97, *p* > 0.05. The mean scores were 86.5 ± 1.1 and 85.1 ± 1.1 for left- and right-handers, respectively.

A musical experience questionnaire was completed by five participants (age: 21.4 ± 4.9 years) who had music training. The questions involved details of the participants’ expertise, including the age at which they started their training (8.6 ± 2.4 years), length of practice (12.8 ± 6.2 years), and the duration of practice/week (12.6 ± 7.3 h). The musicians consisted of percussionists, string and piano players, and self-classified as two left-handers (LI: 4.9 ± 0.1) and three right-handers (LI: 98.5 ± 1.3). The musicians had a time questionnaire index of 84.6 ± 1.8.

### Laboratory Tests

The laboratory tests included six tasks in total: three motor tasks and three time perception tasks. e-Prime software (Psychology Software Tools Inc., Pittsburgh, PA, USA) and PsychoPy software (Peirce, 2007) recorded the responses of the participants in the various tasks. Randomization of performance conditions was conducted within the motor timing and time perception tasks. All scores were averaged over trials per performance condition.

#### Motor Tasks

Finger tapping paradigms enable us to study an individual’s ability for regulating the timing of motor actions. All participants completed the following tasks: paced tapping, unpaced tapping (preferred rate) and unpaced tapping (maximum rate). During paced tapping, the pacing sequence ensures that the task is maintained at a predetermined rate. Such tapping tasks are referred to as externally-guided. During unpaced tapping, the absence of a pacing stimulus permits the participants to self-pace. This type of task is denoted as internally-guided. The tasks were all performed with the index finger of the preferred and non-preferred hand. Position of the fingers/hands was maintained throughout the experiment.

##### Paced tapping

Finger tapping according to a regular or irregular pacing sequence represents an experimental means to study timed responses during sensorimotor synchronization. Participants were required to tap with the index finger in synchrony with auditory tones presented via speakers. The interstimulus interval (ISI) of the pacing sequences was periodically modulated around a baseline interval (1000 ms) using a cosine-wave function, based on evidence that an anticipatory asynchrony can be obtained within an ISI range of 450–1500 ms (Miyake et al., 2004; Serrien and Spapé, 2010). Different magnitudes of timing irregularities were built into the stimulus presentation of the pacing sequences: baseline ± 0%, baseline ± 3%, baseline ± 20%. A trial consisted of a succession of 12.5 basic cycles, with one cycle having the following structure: D, D(1 − A), D, D(1 + A) with D = baseline interval and A = relative perturbation level. Furthermore, the sum of the ISIs in one cycle was equal to 4D. The tones had a duration of 30 ms (including a 15 ms gradual fade-out to prevent tone-offset artifacts) and a pitch frequency of 1000 Hz. Trials lasted 35 s and there were three trials per performance condition. The order of the performance conditions was counterbalanced. After each trial, a subjective report of the regularity of the pacing sequence was obtained by asking the participants whether they had experienced time shifts of the intervals.

##### Unpaced tapping, preferred rate

Finger tapping at preferred tempo measures the internal pace of skill performance, which is spontaneously adopted in daily-life activities. Participants were asked to tap with the index finger as regularly as possible at a comfortable rate that felt subjectively natural. Trials lasted 30 s and there were three trials per hand condition.

##### Unpaced tapping, maximum rate

Finger tapping at maximum speed is an experimental method for contrasting between-hand performances. Participants were instructed to tap with the index finger at their maximum rate. Trials lasted 10 s and there were three trials per hand condition.

#### Time Perception Tasks

Time perception paradigms are used to investigate an individual’s subjective ability to assess intervals of time. All participants were asked to complete the following tasks: verbal production of interval duration, verbal estimation of interval duration, and detection of timing shifts during pacing sequences.

##### Verbal time production

Evaluating the time of a prespecified interval is an experimental means to study time perception. Participants were instructed to verbally evaluate a time interval equivalent to a duration that was previously specified. Therefore, there is a requirement to translate an objectively labeled duration to a subjectively experienced duration. During the task, participants were distracted by watching single digits that appeared about every second on the computer screen in order to prevent subvocal counting. For each trial, participants were instructed to say stop when they thought that an interval of 20, 40 or 60 s had passed in addition to their counts that a particular digit had appeared on the screen. Each time interval was presented three times. No feedback was given to the participants.

##### Verbal time estimation

Estimating the time of an ongoing interval is an additional method to assess time perception. Participants were asked to verbally estimate the duration of a time interval after it had passed. This implies a requirement to translate a subjectively experienced duration to an objectively labeled duration. During the task, participants were distracted by watching single letters that appeared about every second on the computer screen in order to prevent subvocal counting. For each trial, participants were asked to estimate covertly the duration of the elapsed time interval that corresponded to 15, 30 or 45 s in addition to their counts that a specific letter had appeared on the screen. Each time interval was presented three times. No feedback was given to the participants.

##### Detection of time shifts

Evaluating the (ir)regularity of a pacing sequence reveals the perceived temporal structure without the contribution of motor activity. Participants were asked to listen to stimuli sequences with no, subliminal and supraliminal time shifts (baseline interval of 1000 ms ± 0%, ± 3%, ± 20%, respectively) similar to the tapping conditions. A subjective report of the regularity of the sequence was obtained after each trial by asking the participants whether they had experienced time shifts of the intervals.

### Measurements

#### Motor Tasks

##### Paced tapping

The mean intertap interval (ITI) expresses the time of the successive tap responses and provides an indication about the robustness of the internal time dynamics. The coefficient of variation (CV) of the ITI (obtained by dividing the standard deviation by the average ITI) represents the consistency of the successive tap responses. Finally, the percentage of perceived regularity of the tones reflects the subjective experience of the pacing sequence during tapping.

##### Unpaced tapping (preferred and maximum rate)

The ITI (mean and CV) was calculated to represent the accuracy and variability of the timing performances.

#### Time Perception Tasks

##### Verbal time production

The time production ratio (the perceived time for each interval divided by the actual duration of the interval) was calculated, providing a measure of produced vs. actual interval duration. A score of less/greater than 1 represents an underproduction/overproduction of the time duration. The average time production ratio and the CV of the produced times were computed. Furthermore, the digit count ratio (the perceived counts of a digit in each interval divided by the actual counts of the digit per interval) was determined. The average digit count ratio and the CV of the participants’ digit counts were calculated.

##### Verbal time estimation

The time estimation ratio (the estimated time for each interval divided by the actual duration of the interval) was calculated, giving a measure of estimated vs. actual interval duration. A score of less/greater than 1 represents an underestimation/overestimation of the time duration. The average time estimation ratio and the CV of the estimated times were calculated. Also, the letter count ratio (the perceived counts of a letter in each interval divided by the actual counts of the letter) was established. The average letter count ratio and the CV of the participants’ letter counts were computed.

##### Detection of time shifts

The percentage of perceived regularity of the tones was calculated, representing the subjective experience of the pacing sequences during listening (without tapping).

### Analysis

Different types of analyses were conducted for the motor timing and time perception tasks and involved mixed-design ANOVAs as well as correlation analyses. Mean ± SE scores are reported. *Post hoc* comparisons with Bonferroni corrections were made where necessary.

#### Motor Tasks

For paced tapping, the ITI measurements (mean, CV) in addition to the percentage of perceived regularity of the pacing sequence were analyzed by means of 2 (Group; left- and right-handers) × 3 (Perturbation condition; no = 0%, subliminal = 3% and supraliminal = 20%) × 2 (Hand; left and right) mixed ANOVAs. The between-subject factor was Group whereas the within-subject factors were Perturbation condition and Hand. For unpaced tapping (preferred and maximum rate), the ITI measurements were analyzed according to 2 (Group; left- and right-handers) × 2 (Hand; left and right) mixed ANOVAs. The between-subject factor was Group whereas the within-subject factor was Hand.

#### Time Perception Tasks

The time production ratio, time estimation ratio and count ratio measurements (mean, CV) were analyzed by means of 2 (Group; left- and right-handers) × 3 (Interval condition; short, intermediate and large) mixed ANOVAs. The between-subject factor was Group whereas the within-subject factor was Interval condition. In addition, the percentage of perceived regularity of the pacing sequences were analyzed by means of 2 (Group; left- and right-handers) × 3 (Perturbation condition; no = 0%, subliminal = 3% and supraliminal = 20%) × 2 (Hand; left and right) mixed ANOVAs. The between-subject factor was Group whereas the within-subject factors were Perturbation condition and Hand. In addition, Pearson correlation coefficients were calculated between: (1) the time production ratio and motor tapping rates; (2) the time production/estimation ratio and time questionnaire index; and (3) the time production ratio and time estimation ratio.

## Results

### Motor Tasks

#### Paced Tapping: ITI

**Figure 1:**
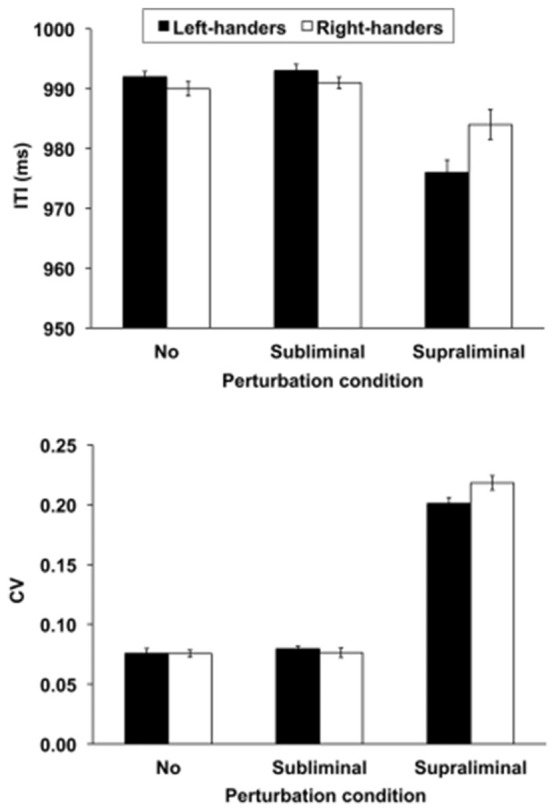
The mean intertap interval (ITI, upper panel) and coefficient of variation (CV, lower panel) for the left- and right-handers as a function of perturbation condition, i.e., no = 0%, subliminal = 3% and supraliminal = 20%. The mean ± SE scores are illustrated.

The mean ITI revealed a significant main effect of Perturbation condition, *F*_(2,72)_ = 23.39, *p* < 0.01, and a significant Group × Perturbation condition interaction, *F*_(2,72)_ = 3.58, *p* < 0.05 (Figure 1, upper panel). This interaction pointed out that the handedness groups did not show ITI differences in the no and subliminal perturbation conditions that involved temporal stability whereas the left- as compared to right-handers performed better in the supraliminal perturbation conditions that required temporal flexibility, i.e., they were less close to the baseline interval of 1000 ms, *p* < 0.05. These findings suggest that left-handers adapted more effectively to the supraliminal time shifts than right-handers who performed more tightly to the baseline interval. No other effects were significant, *p* > 0.05. A follow-up correlation analysis conducted separately for the left- and right-handers between the participants’ LI and the supraliminal perturbation ITI scores did not reveal significant effects, *p* > 0.05. This result suggests that the degree of handedness of both groups did not affect the ITI responses.

The CV of the ITI showed a significant main effect of Perturbation condition, *F*_(2,72)_ = 778.4, *p* < 0.01, and a significant Group × Perturbation condition interaction *F*_(2,72)_ = 6.48, *p* < 0.01 (Figure 1, lower panel). Although the tap responses were less consistent in the supraliminal conditions than in the other task conditions, right-handers were more variable than left-handers in their responses when supraliminal perturbations were encountered (*p* < 0.05), while the other conditions did not differ from one another, *p* > 0.05. No other effects were significant, *p* > 0.05. A follow-up correlation analysis conducted separately for the left- and right-handers between the participants’ LI and the supraliminal perturbation CV scores did not show significant effects, *p* > 0.05. This finding shows that the degree of handedness did not influence the consistency of the tap responses.

#### Paced Tapping: Subjective Report

The percentage of perceived regularity revealed a significant main effect of Perturbation condition, *F*_(2,72)_ = 106.0, *p* < 0.01. Perceived regularity was not different in the no and subliminal perturbation conditions (*p* > 0.05) whereas both differed from the supraliminal perturbation conditions, *p* < 0.05. The mean percentage scores were 98.3 ± 1.1%, 92.6 ± 2.2% and 26.9 ± 5.8% for the no, subliminal and supraliminal perturbation conditions, respectively. No other effects were significant, *p* > 0.05.

#### Unpaced Tapping, Preferred Rate: ITI

The mean ITI demonstrated no significant effects, *p* > 0.05. The mean scores for the left and right hand were 525.1 ± 40.5 ms and 526.0 ± 41.5 ms across handedness groups.

The CV of the ITI showed no significant effects, *p* > 0.05. The mean scores for the left and right hand were 0.092 ± 0.009 and 0.087 ± 0.009 across handedness groups.

#### Unpaced Tapping, Maximum Rate: ITI

The mean ITI presented a significant Group × Hand interaction, *F*_(1,36)_ = 40.74, *p* < 0.01. This interaction revealed that both handedness groups moved faster with their preferred as compared to non-preferred hand, *p* < 0.05. The mean scores for the left and right hand were 152.4 ± 3.9 ms and 161.2 ± 3.3 ms for left-handers, 162.1 ± 4.2 ms and 146.7 ± 3.6 ms for right-handers. No other effects were significant, *p* > 0.05.

The CV of the ITI indicated no significant effects, *p* > 0.05. The mean CV scores for the left and right hand were 0.27 ± 0.03 and 0.25 ± 0.03 for left-handers, 0.23 ± 0.02 and 0.26 ± 0.04 for right-handers.

### Time Perception Tasks

#### Verbal Time Production: Time Production Ratio and Digit Count Ratio

The data of the verbal time production tasks are presented in Table 2.

**Table 2:** The data from the time production and time estimation tasks for the left- and right-handers.

	Time production task				
Left-handers measure	Ratio	CV		Ratio	CV
Time production			Digit count	
Short	1.359 ± 0.081	0.175 ± 0.031	Short	0.977 ± 0.020	0.156 ± 0.030
Intermediate	1.292 ± 0.047	0.155 ± 0.028	Intermediate	0.988 ± 0.024	0.150 ± 0.029
Long	1.244 ± 0.054	0.089 ± 0.019	Long	1.053 ± 0.026	0.076 ± 0.015
**Right-handers measure**	**Ratio**	**CV**		**Ratio**	**CV**
**Time production**			**Digit count**	
Short	1.341 ± 0.068	0.147 ± 0.025	Short	0.997 ± 0.019	0.168 ± 0.029
Intermediate	1.266 ± 0.059	0.133 ± 0.027	Intermediate	1.018 ± 0.028	0.162 ± 0.030
Long	1.230 ± 0.066	0.101 ± 0.022	Long	1.029 ± 0.033	0.088 ± 0.016
	**Time estimation task**				
**Left-handers measure**	**Ratio**	**CV**		**Ratio**	**CV**
**Time estimation**			**Letter count**	
Short	0.890 ± 0.058	0.152 ± 0.037	Short	1.000 ± 0.003	0.006 ± 0.004
Intermediate	0.826 ± 0.082	0.111 ± 0.023	Intermediate	0.989 ± 0.007	0.016 ± 0.008
Long	0.800 ± 0.063	0.118 ± 0.029	Long	1.006 ± 0.018	0.009 ± 0.005
**Right-handers measure**	**Ratio**	**CV**		**Ratio**	**CV**
**Time estimation**			**Letter count**		
Short	0.912 ± 0.092	0.134 ± 0.026	Short	1.000 ± 0.005	0.010 ± 0.009
Intermediate	0.854 ± 0.087	0.105 ± 0.028	Intermediate	1.003 ± 0.007	0.022 ± 0.011
Long	0.844 ± 0.079	0.130 ± 0.032	Long	1.014 ± 0.025	0.015 ± 0.006

*The time production/estimation ratio, digit/letter count ratio alongside the coefficient of variation (CV) for the different time intervals: short, intermediate and long. Mean ± SE scores are illustrated*.

The mean time production ratio illustrated a significant main effect of Interval condition, *F*_(2,72)_ = 3.34, *p* < 0.05. Participants overproduced the duration of the intervals, which reduced as the length of the interval increased. Moreover, the short interval differed from the long interval, *p* < 0.05. No other effects were significant, *p* > 0.05.

The CV of the produced time revealed a significant main effect of Interval condition, *F*_(2,72)_ = 5.23, *p* < 0.01. The variability for the short interval differed from the long interval, *p* < 0.05. No other effects were significant, *p* > 0.05.

The mean digit count ratio showed no significant effects, *p* > 0.05.

The CV of the digit count demonstrated a significant main effect of Interval condition, *F*_(2,72)_ = 4.62, *p* < 0.01. The variability was highest for the short interval which differed from the long interval, *p* < 0.05. No other effects were significant, *p* > 0.05.

**Figure 2:**
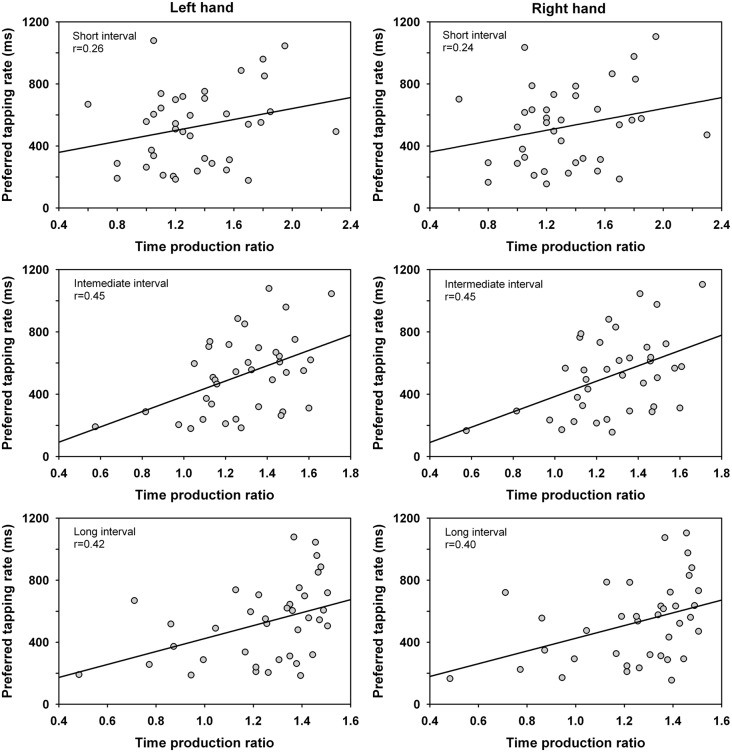
Correlations between the time production ratio for the short interval (20 s), intermediate interval (40 s), and long interval (60 s) and the preferred tapping rate of the left and right hand. The time production ratio provides a measure of the participants’ produced vs. actual interval duration whereas the preferred tapping rate measures the spontaneous tempo adopted under unpaced conditions.

Correlation coefficients were calculated between the time production ratio and the preferred tapping rate of the left and right hand for the different intervals. The analysis was conducted across groups as handedness did not affect the time production ratio nor the preferred tapping rate. The analysis showed significant positive correlations for the intermediate and long interval durations (i.e., the 40–60 s temporal range), *p* < 0.01. This finding suggests an association between produced motor timing and time perception when driven by natural self-pacing for a sufficient duration of time. The correlation coefficients were *r*_(36)_ = 0.26, 0.45, 0.42 (left hand), and 0.24, 0.45, 0.40 (right hand) for the short, intermediate and long interval, respectively (Figure 2). Of note is that one participant showed rather extreme values with a fast preferred tapping rate and low time production ratio (evaluating the duration length shorter than it is). When the scores of this participant were eliminated from the analyses, significant positive correlations continued to be observed for the intermediate and long intervals. The correlation coefficients for the intermediate and long interval were *r*_(35)_ = 0.41 and 0.37 (left hand), *r*_(35)_ = 0.39 and 0.34 (right hand), *p* < 0.05. No significant correlations of the time production ratio were observed with the timing of paced tapping or unpaced tapping at maximum rate, *p* > 0.05.

**Figure 3:**
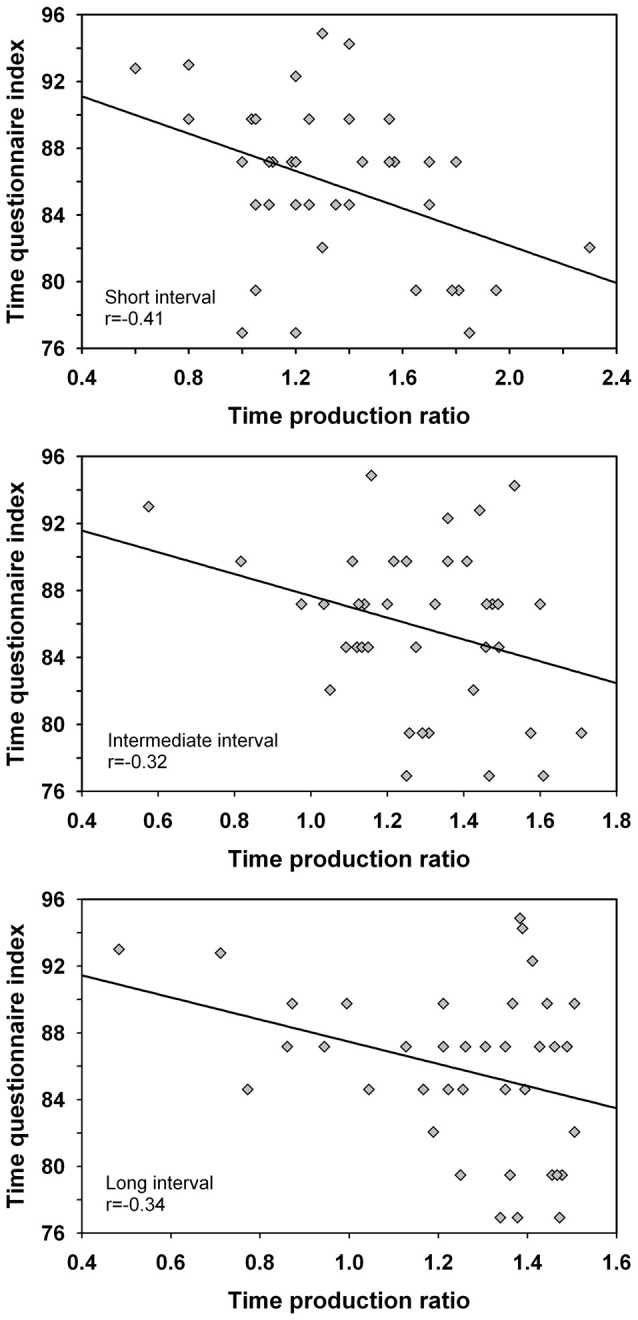
Correlations between the time production ratio for the short interval (20 s), intermediate interval (40 s) and long interval (60 s) and the ratings of the passage of time as established by the time questionnaire. Only the correlation for the short interval was significant when the extreme scores of one participant were eliminated from the analyses.

Correlation coefficients were computed between the time production ratio and the time questionnaire index for the different intervals. The analysis was made across groups as handedness did not influence the time production ratio nor the time questionnaire index. The analysis suggested significant negative correlations for the short interval *r*_(36)_ = −0.41 (*p* = 0.01), intermediate interval *r*_(36)_ = −0.32 (*p* < 0.05) and long interval *r*_(36)_ = −0.34 (*p* < 0.05) such that overproduced interval durations associated with smaller ratings of the passage of time i.e., a decelerated subjective time (Figure 3). When the extreme scores of one participant with a low time production and high rating of the passage of time were eliminated from analyses, only the correlation for the short duration remained significant (i.e., the 20 s duration length), *r*_(35)_ = −0.36 (*p* < 0.05). Therefore, more research is required to investigate the robustness of the finding and to validate the association between both variables.

#### Verbal Time Estimation: Time Estimation Ratio and Letter Count Ratio

The data of the verbal time estimation tasks are shown in Table 2.

The time estimation ratio indicated a significant main effect of Interval condition, *F*_(2,72)_ = 3.30, *p* < 0.05. Participants underestimated the duration of the intervals, which became stronger as the length of the interval increased. In particular, the short interval differed from the long interval, *p* < 0.05. No other effects were significant, *p* > 0.05.

The CV of the estimated time did not reveal significant effects, *p* > 0.05.

The letter count ratio in addition to the CV of the letter count showed no significant effects, *p* > 0.05.

**Figure 4:**
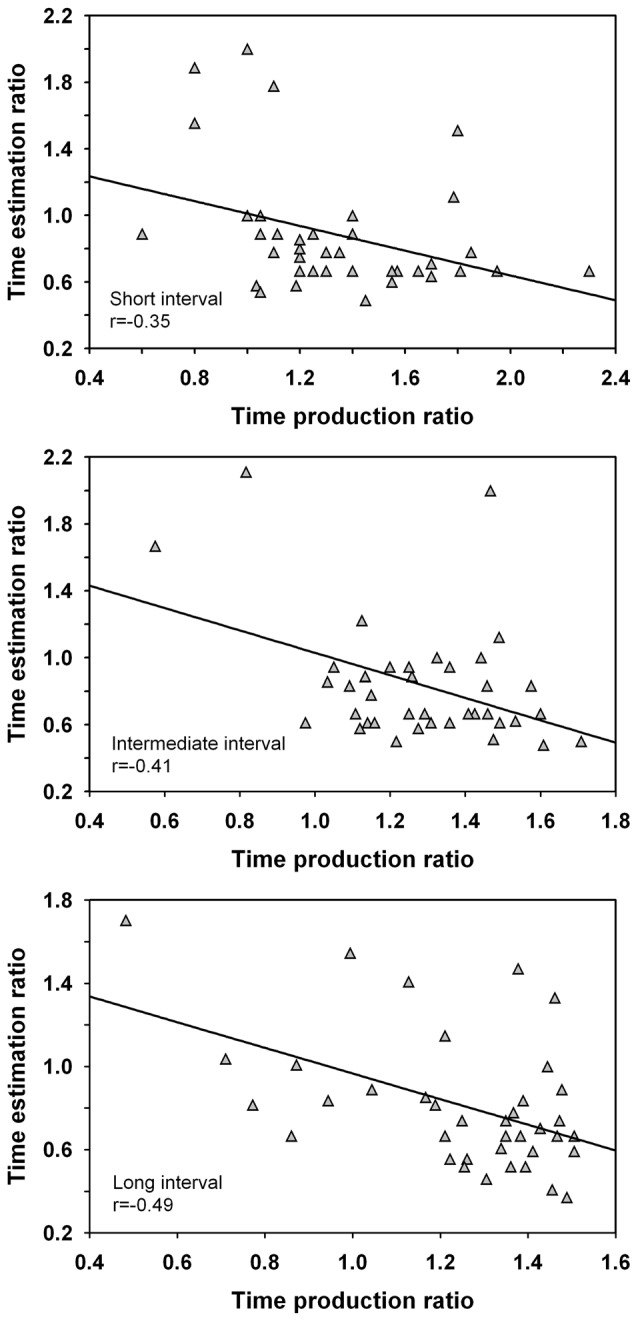
Correlations between the time estimation ratio and time production ratio for the short, intermediate and long interval. The time estimation ratio gives a measure of the participants’ estimated vs. actual interval duration whereas the time production ratio provides a measure of the participants’ produced vs. actual interval duration. Only the correlation for the long interval was significant when the extreme scores of one participant were eliminated from the analyses.

Correlation coefficients were calculated between the time estimation ratio and the time production ratio. The analysis was conducted across groups as handedness did not impact on the time estimation ratio nor on the time production ratio. The analysis revealed significant negative correlations between the short intervals, *r*_(36)_ = −0.35, *p* < 0.05, intermediate intervals, *r*_(36)_ = −0.41, *p* = 0.01, and long intervals, *r*_(36)_ = −0.49, *p* < 0.01, and are shown in Figure 4. This result suggests a negative coupling between both time perception tasks and hints at the involvement of different processing resources. However, a number of extreme scores were observed across the intervals. When the extreme scores of one participant with a high time estimation ratio and low time production ratio for the long interval were removed from the analyses, a significant negative correlation remained present for this duration length (i.e., >40 s temporal range), *r*_(35)_ = −0.32, *p* < 0.05, but not for the intermediate interval, *p* > 0.05. However, the result needs to be interpreted with caution and the reliability of this finding requires further investigation. No significant correlations of the time estimation ratio were noted with the timing of paced or unpaced tapping, or with the time questionnaire, *p* > 0.05.

#### Listening: Subjective Report

The percentage of perceived regularity showed a significant main effect of Perturbation condition, *F*_(2,72)_ = 69.3, *p* < 0.01. The mean percentage scores were 91.3 ± 3.2%, 90.3 ± 3.3% and 28.0 ± 5.9% for the no, subliminal and supraliminal perturbation conditions, respectively. No other effects were significant, *p* > 0.05.

## Discussion

Throughout development we acquire a sense of duration and rhythm that is an integral part of everyday activities such as speaking or dancing to music (Kirschner and Tomasello, 2009). In particular, we rely on timekeepers such as cues to track temporal regularity that results in expectation and the ability to predict future events; an ability also referred to as temporal extrapolation (Jazayeri and Shadlen, 2010; Bendixen et al., 2012; Schwartze and Kotz, 2015). This predictive ability further allows us to subjectively experience time (Bechara et al., 1996). In the present experiment, we have studied motor timing and time perception tasks with a particular focus on handedness; a defined form of individual differences that is associated with behavioral and neural distinctiveness. The investigation offers an enhanced understanding about domain-general and domain-specific processing that support timed behavior during everyday activities. Here, we have used two complementary approaches: (1) questionnaires that enabled participants to rate their abilities and preferences; (2) experimental tasks that provided behavioral accuracy and consistency data.

### Motor Timing and the Influence of Handedness

The ability to interact in the environment relies on the coupling between perception and action (sensorimotor timing) supported by error correction mechanisms (Repp, 2005); a skill that has experimentally been studied by means of finger tapping tasks during which participants tap in synchrony with a pacing sequence. In this case, timing is guided externally and predicting the time of the upcoming events strengthens the behavioral responses and facilitates the task goal (Coull and Nobre, 2008; Serrien, 2008). Previous work has shown that the temporal aspects of this task are controlled by distributed clusters across the basal ganglia, cerebellum and premotor-parietal circuitry (Rao et al., 1997; Jäncke et al., 2000; Pollok et al., 2006). However, the brain areas that regulate the timing demands are flexibly organized and influenced by task-related factors such as the pacing sequence, timescale and assignment complexity (Witt et al., 2008).

The data from the ITIs revealed that the participants performed differently as a function of the perturbation condition. In particular, their timed responses were similar during the no and subliminal perturbation conditions whereas they deviated more from the base interval of 1000 ms during the supraliminal perturbation conditions. These observations are in line with research that has shown that irregular pacing sequences with time shifts that are below the perceptual threshold are not differently processed than regular pacing sequences (Aschersleben, 2002; Repp, 2005; Serrien and Spapé, 2010). Conversely, irregular pacing sequences with supraliminal perturbations implicate a more flexible processing mode. The previous statement proposes that the (ir)regularity of the pacing sequence determines the level of processing; a premise that is supported by brain imaging work. In particular, the neural activation patterns are similar for regular and irregular subliminal conditions whereas irregular supraliminal conditions involve additional activation within cerebellum, frontal and parietal regions (Bijsterbosch et al., 2011).

We further observed that left- and right-handers differed in their ability to deal with the supraliminal perturbations that required flexibility as opposed to stability. In particular, right-handers maintained more closely the baseline interval (as in the no and subliminal conditions) and were more variable in their tap responses than left-handers. This finding suggests that left- as compared to right-handers showed an enhanced degree of adaptability in managing effectively perturbative situations that involved cognitive flexibility. The argument is in line with previous research that has shown that left-handers experience less interference than right-handers in cognitive tasks that involve inhibition of overlearned patterns and management of new knowledge (Beratis et al., 2013). Thus, the direction of handedness guides motor control abilities in contextual situations that particularly necessitate cognitive intervention, which is in agreement with the dynamic nature of lateralized brain functions and the expression of dominance in skill use (Serrien et al., 2006). A superior performance of cognitive-motor functions for left- than right-handers has been interpreted to result from greater communication across hemispheres and/or increased access to right hemisphere processes (Beratis et al., 2013), which allows updating in response to changing demands. Increased hemispheric communication is based on the idea that it facilitates transfer of information between sides, or, allows one hemisphere to inhibit processing in the other (Chiarello and Maxfield, 1996). Further neuroimaging studies would be required to detail the neural basis of the group differences and aspects of timing performance.

In contrast to the performance scores of paced tapping, the perceived regularity of the pacing sequences showed no differences between handedness groups. This implies that the subjective impression of the occurring stimuli was similar for both left- and right-handers. These data from the subjective reports extend previous observations (Aschersleben, 2002) that participants independent of their handedness do not notice subliminal perturbations during pacing sequences but they do when encountering supraliminal perturbations.

In addition to paced tapping, we also evaluated unpaced tapping in order to assess individual variation in the internal regulation of preferred and maximum timing rates. The results demonstrated that preferred tapping rates were subject to individual differences with time intervals averaging around 500–600 ms, which is in accordance with earlier research (Fraisse, 1982; Collyer et al., 1994; Delevoye-Turrell et al., 2014). Overall, there is agreement that the preferred tempo we adopt across motor activities is a comfortable one that supports efficient timing within an individual time range (van Noorden and Moelants, 1999). Thus, even though the preferred tempo shows high inter-individual variability, this is opposed to relatively low intra-individual variability. The latter premise is supported by our finding that the preferred tapping rate of the left and right hand associated with the time production tasks that required participants to internally generate a designated interval duration and identify when they thought the time had lapsed. In particular, a positive correlation was observed between the produced motor timing assessed over a prolonged time interval and time perception tasks for the intermediate and long interval that comprised the 40–60 s temporal range under investigation. Such stability of an individual’s timing ability suggests the existence of common mechanisms across domain for the processing of interval duration. Therefore, cross-domain coupling may occur when there is reliance on rates that are spontaneously adopted for motor behavior and for those we perceive. In particular, both timing skills can be considered as counterparts that share an intrinsic (preferred) timescale and that take advantage of interwoven domain-general mechanisms to support performance. The finding is in line with brain imaging data that have demonstrated that unpaced tapping particularly engages the medial premotor system, including the supplementary motor area (SMA), putamen, thalamus in addition to inferior frontal cortex (Rao et al., 1997), and internally generated time intervals preferentially involve SMA and right-sided prefrontal circuitry (Wiener et al., 2010). These observations underline that both timing skills may be supported by core neural correlates such as medial premotor areas that operate across domain.

In contrast to similar preferred tapping rates across hands and handedness groups, the data from the maximum speed rates showed distinct effects. In particular, the fastest tempo was observed for the preferred as compared to non-preferred hand for left- and right-handers. This result indicates an asymmetrical performance difference that is driven by sensorimotor mechanisms and confirms that speed tapping represents an objective measurement of handedness. Taken together, our findings agree with previous work that has shown that between-hand asymmetries vary as a function of tapping rate with differences being present for tapping at fastest speed, while reduced at fast steady rate and absent at slow rate (Truman and Hammond, 1990).

It is noteworthy that although the left-handers as a group involved consistent and inconsistent handers, this was not so for the right-handers for which only one participant could be classified as an inconsistent hander. In this respect, a strongly handed person consistently uses either their left or right hand for manual activities whereas less strongly handed individuals show less consistent hand preferences. Therefore, inconsistent left-handers are typically more common than inconsistent right-handers. This argument is supported by findings that have revealed that right- as compared to left-handers usually exhibit a stronger hand preference in handedness inventories, likely because of the use of their preferred hand most of the time (Gurd et al., 2006; Bryden et al., 2007). In addition, five participants in our study had music training. However, the expertise did not affect their handedness preferences for daily-life activities as the musicians consisted of left- and right-handers with rather extreme laterality scores, i.e., individuals who could be labeled as consisted handers.

### Time Perception and its Different Components

The sense of time is known to be an essential element of our everyday behavior and decision-making (Merchant et al., 2013). Subjective time involves at least two components; judgement of the passage of time (i.e., how fast time seems to pass) and the evaluation of duration length (i.e., how long an event seemed to have lasted), (Wearden, 2015). However, these temporal experiences can vary independently of one another, resulting in a dissociation of both such as when slow passage of time is associated with duration estimations that are shorter than they actually are (Weiner et al., 2016). To investigate both components, we first asked the participants to answer a questionnaire in order to obtain an index of how they subjectively experience time (Sucala et al., 2010). The questions related to naturalistic situations that guide the perceived speed of time. For example, individuals who are less active tend to feel as if time is passing more slowly such as in situations when one is bored (Zakay, 2014). This means that as the time dimension becomes relevant, intervals are perceived as longer as opposed to when intervals are filled with activities that distract from attending to time (Wittmann, 2013; Duzcu and Hohenberger, 2014). Second, in order to evaluate the perception of interval length, we assessed the participants in two different paradigms during which they had to produce and estimate the duration of a range of time intervals. During verbal time production, participants were required to specify the duration of intervals. Therefore, one evaluates time as it is passing, relying strongly on attention-demanding processes while recall only plays a minor role. Conversely, during verbal time estimation, participants were asked to judge a time interval after it has passed such that recall is an essential process with reliance on events that occurred during the interval (Grondin, 2010; Sucala et al., 2010). In our data analysis, we did not report on the scalar properties of timing as our temporal tasks did not include performance-related feedback, which plays a significant role in supporting the relationships as specified by scalar timing (Wearden and Lejeune, 2008).

Our results showed that the majority of the participants were relatively close to the different interval durations in all tasks but overproduced (interval length is evaluated longer than it is) and underestimated (interval length is estimated shorter than it is) the time in relation to the set targets. That left- and right-handers did not show differences in the perception of time intervals suggests domain-general mechanisms that are not driven by the neural representational changes due to handedness. Additional analysis suggested that time production and time estimation measurements correlated with one another in opposite directions, pointing to different evaluation processes with a specific involvement of attention to time and memory for events, respectively. The inverse relationship between the duration judgements obtained for time production and time estimation has been proposed previously (Zakay and Block, 2004). However, our finding was only reliable for the long interval (>40 s temporal range) but not for the short and intermediate intervals. In this respect, it has been argued that the sensitivity of time perception depends on the particular circumstances in which the time intervals are experienced (Matthews and Meck, 2014) with several factors guiding the amount of cognitive resources allocated to temporal processing such as the cognitive load of the task (Zakay and Block, 2004). Therefore, it is possible that the cognitive demands across both tasks more closely matched in the longest time intervals than in the short and intermediate time intervals. However, more research is required to examine the reliability of the finding.

Furthermore, the time production task negatively correlated with the time questionnaire ratings such that overproduced interval durations associated with a slowing down of the passage of time. The result that was only reliable for the short interval (20 s duration length) but not for the longer intervals proposes a relation between individual time perception and the subjective sense of time passing in everyday life. In this regard, previous research has proposed that temporal relevance affects both the estimates of interval length and passage of time judgments (Zakay and Block, 2004; Sucala et al., 2010). Thus, it is feasible that time awareness most closely corresponded for the short interval under investigation. However, further work is needed to investigate the reliability of the result and the dynamic nature of the association between both time experiences. Together, the data illustrate the value of combining different measures of timing and interval durations. Prior research on time perception has revealed a strong sensitivity of temporal judgments of intervals to the task requirements, contextual factors and individual differences, reflecting the plastic and dynamic nature of temporal representations (Matthews and Meck, 2014). These dependencies complicate the investigations to evaluate regularities and to provide unified approaches of time perception performances. Examining the coupling of timing activities can accordingly provide a rich source of information that has relevance for the understanding of human behavior and diversity.

Handedness did not influence the time perception measurements in our study. Previously, Vicario et al. (2011) examined left- and right-handers who responded with the left or right hand during a time reproduction task of sub-second and supra-second time intervals. Their results showed that left-handers underestimated the intervals that required supra-second processing, independent of hand used. This suggests that a time perception task accompanied by a motor response of either hand can be driven by handedness, likely due to the involvement of motor circuitry in temporal processing. It further indicates that temporal computations rely on the predictive power of the motor system (Schubotz et al., 2000). Hence, it can be argued that contextual and task demands steer the effect of handedness in time perception tasks.

In conclusion, time is an important dimension that supports many activities in everyday life. The present study showed covariation across left- and right-handers between movement-driven timing and perceived interval duration when participants adopted their preferred tempo. Handedness guided motor timing during externally-driven conditions that required cognitive intervention. Overall, our results highlight that individual factors through domain-general and domain-specific levels of organization play a steering role in how one predicts, perceives and experiences time, which accordingly impacts on cognition and behavior.

## Author Contributions

LOR, MMS and DJS designed the study. LOR collected the data, DJS analyzed the data. LOR, MMS and DJS drafted and revised the manuscript. All authors approved the final version of the manuscript.

## Conflict of Interest Statement

The authors declare that the research was conducted in the absence of any commercial or financial relationships that could be construed as a potential conflict of interest.
