# Supplementary material for: Motor Timing and Covariation with Time Perception: Investigating the Role of Handedness
Source: Front Behav Neurosci. 2017 Aug 15;11:147. doi: 10.3389/fnbeh.2017.00147 (PMC5559439; doi:10.3389/fnbeh.2017.00147)
Supplement: Supplementary file 1 [file Data_Sheet_1.docx]

Appendix 1. Handedness questionnaire

1. Write a letter

2. Use spoon to eat soup

3. Use scissors when cutting paper

4. Use toothbrush

5. Throw a ball to hit a target

6. Hold a racquet when playing tennis

7. Use eraser on paper

8. Hold needle when sewing

9. Use a comb

10. Deal cards

11. Use key to unlock door

12. Peel an apple

13. Unscrew lid from jar

14. Flip a coin

15. Use a knife to cut bread

16. Draw a picture

17. Use a broom for sweeping (upper hand)

18. Open lid from drink can

19. Use a bottle opener

20. Use a computer mouse

Appendix 2: Time questionnaire

1. I often feel time pressure

2. My life is filled with many new experiences

3. When I am waiting, I feel time passes slowly

4. These days, I am not very busy

5. I often feel bored

6. It upsets me to be late for appointments

7. I worry if things do not get done on time

8. Time flies by

9. Being able to organise my activities in time is important for me

10. I frequently have to rush to make sure everything gets done

11. I often find that events happened much longer ago than I thought

12. When I am doing a pleasant activity, I feel time passes more quickly

13. I have to establish my priorities to do all the things I would like to do
